# Massive Gastric Juvenile Polyposis Associated With Intermittent Gastric Outlet Obstruction: A Case Report

**DOI:** 10.7759/cureus.61792

**Published:** 2024-06-06

**Authors:** Dimitrios Linardoutsos, Evgenia Mela, Stamatina Triantafyllou, Anastasios Machairas

**Affiliations:** 1 First Propaedeutic Department of Surgery, Hippocration General Hospital, National and Kapodistrian University of Athens, School of Medicine, Athens, GRC; 2 Department of Surgery, Metropolitan General Hospital, Athens, GRC

**Keywords:** management, total gastrectomy, smad4 mutation, massive gastric juvenile polyposis, juvenile polyps

## Abstract

Juvenile polyposis syndrome is a condition distinguished by numerous hyperproliferative polyps that can affect the entire gastrointestinal tract, though they are uncommon in the stomach. We report a rare case of a 70-year-old woman with a three-year history of epigastric pain and severe bloating who was referred to our department for gastric outlet obstruction due to massive gastric juvenile polyps also causing gastroparesis. The patient was successfully treated with a total gastrectomy.

## Introduction

Juvenile polyposis syndrome is an uncommon clinical entity inherited by the autosomal dominant pattern. The referred incidence ranges from 1/100,000 to 1/160,000 [1]. Multiple hamartomatous polyps of the entire gastrointestinal tract are the most prominent clinical signs [1]. Up to 60% of patients exhibit SMAD4 or BMPR1A germline mutations [2]. The most commonly affected sites are the colon and rectum (98%), followed by the stomach (14%), and the small bowel (14%) [1]. We present an unusual case of a 70-year-old woman diagnosed with massive gastric polyposis in the context of juvenile polyposis syndrome.

## Case presentation

A 70-year-old woman was diagnosed with juvenile polyposis syndrome ten years earlier and had regular gastrointestinal surveillance every two years. Upon her presentation, she had three years of intermittent upper abdominal pain, severe nausea, and excessive bloating. On her clinical examination, no significant clinical evidence of peritoneal signs was revealed, other than a palpable abdominal mass in the epigastrium, caused by an extensively distended stomach.

Her medical history was remarkable for right colectomy two years ago due to anemia of polyposis etiology. The specimen’s histopathology report confirmed the existence of multiple juvenile polyps, the largest of which was compatible with the diagnosis of tubulovillous adenoma of low-grade dysplasia. Her family history was negative for other gastrointestinal malignancies. Genetic evaluation was performed and revealed the pathological mutation c.1447+2 T>G / IVS10+2 G>T, located in intron 10 of the SMAD4 gene, which is also carried by other members of her family.

**Figure 1:**
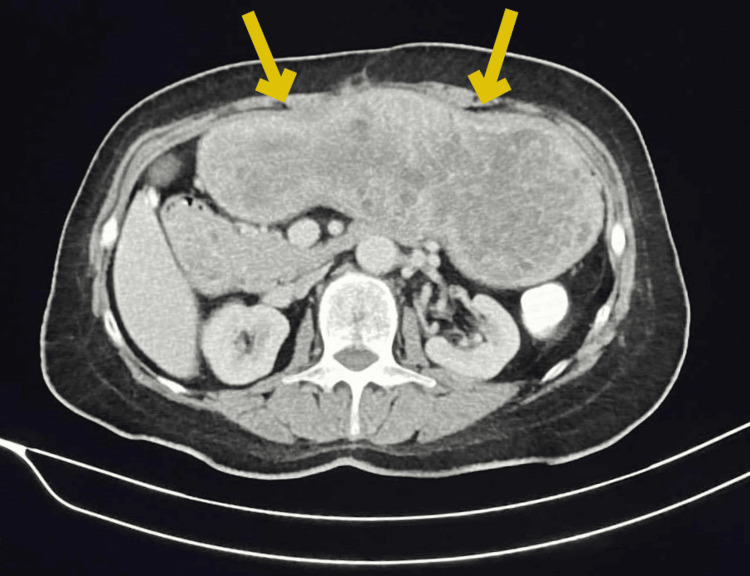
Abdominal CT scan of the distended stomach

**Figure 2:**
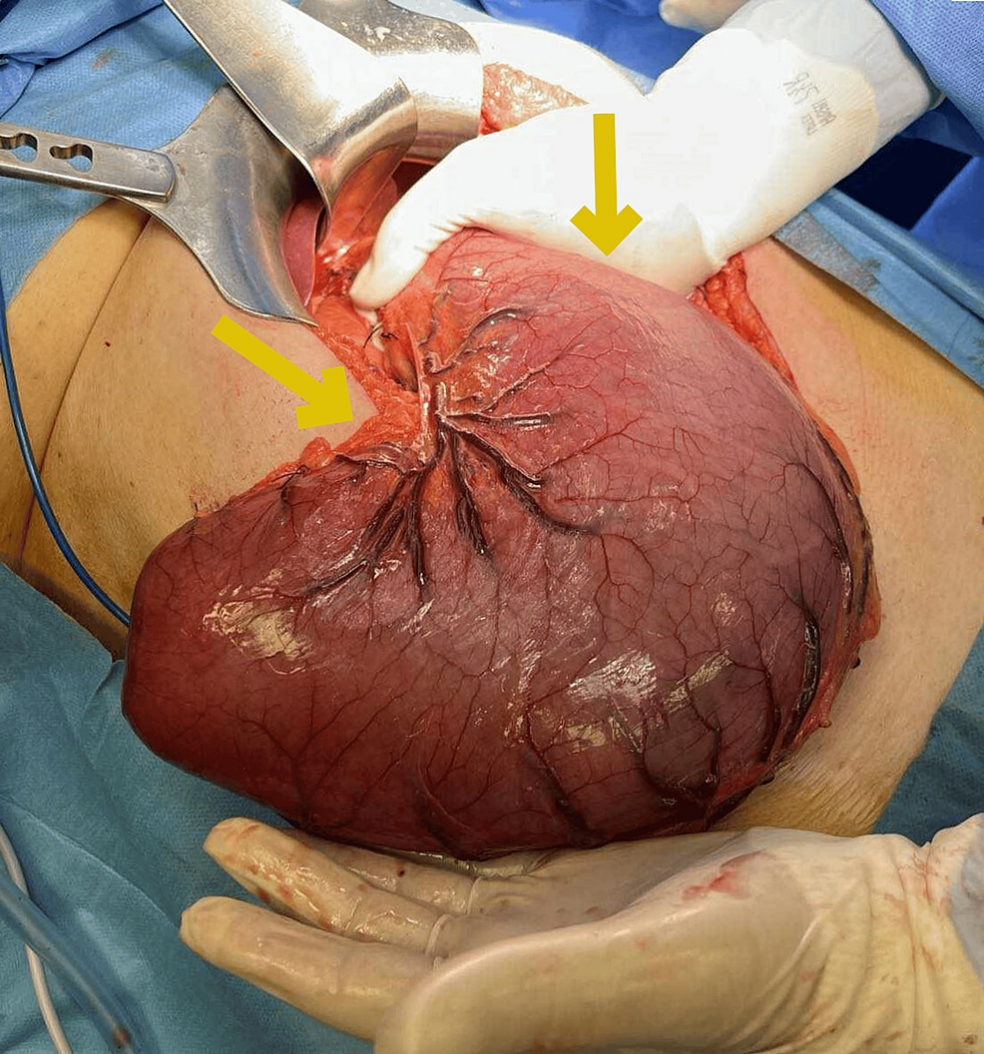
Intraoperative image of the enlarged stomach after mobilization

**Figure 3:**
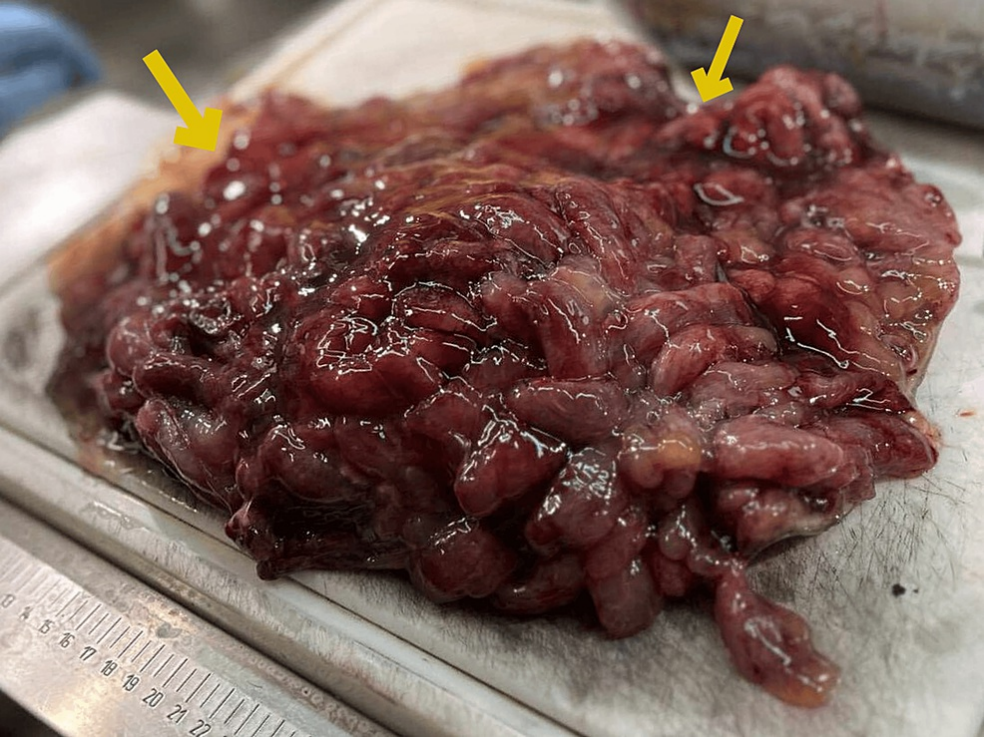
Macroscopic image of the numerous juvenile polyps occupying diffusely the lumen of the excised stomach

Upon evaluation, laboratory diagnostic tests were normal, except for iron deficiency anemia. On esophagogastroduodenoscopy, massive gastric polyposis was obvious, with a big polyp with a maximum length of 1.8 cm, causing intermittent gastric outlet obstruction and compromising the motility of the stomach due to progressive distension, thus causing gastroparesis. A CT scan yielded a distended stomach with numerous polyps (Figure 1). Clinically and with genetic research, the diagnosis of gastric juvenile polyposis was clear, and given the recurrent gastric outlet obstruction and gastroparesis, surgical excision of the stomach was recommended. Subsequently, the patient underwent a total gastrectomy with D1 lymph node dissection, due to the absence of evidence of malignancy from the CT scan and biopsy, and a 50-cm Roux-en-Y reconstruction. Intraoperatively, the distended stomach occupied the whole upper abdomen, reaching 10 cm below the umbilicus (Figure 2). The resected specimen length measured 61 cm along the greater curvature and 16 cm along the lesser curvature. Histopathological examination revealed numerous, more than 50, polypoid lesions with edema and inflammatory infiltration of the lamina propria, epithelial hyperplasia, and cystic dilatation of glands (Figure 3). In addition, ulceration with the formation of inflammatory granular tissue and, in other locations, low-grade dysplasia were observed. Thus, the final histopathology report of the resected specimen confirmed massive juvenile polyposis affecting the stomach. The postoperative course was unremarkable, and the patient was discharged six days postoperatively, having an uneventful recovery.

## Discussion

Juvenile polyposis syndrome (JPS) is a rare disorder characterized by numerous hyperproliferative polyps in the gastrointestinal tract [1]. Although in most newly diagnosed cases (75%), it is an autosomal dominantly inherited disorder for patients with a positive family history, it can be sporadic in 25% of cases appearing due to de novo mutations in the genes that have been implicated [1,3]. Polyps begin to arise during the first decade of life, and the median age of diagnosis is 18.5 years [1]. The diagnosis of JPS is reached when any one of the three criteria occurs (Jass criteria): five or more juvenile polyps of the colon, and/or multiple juvenile polyps throughout the gastrointestinal tract, and/or juvenile polyps of any number in patients with a family history of juvenile polyposis [4]. The incidence of juvenile gastric polyposis is considered to be undervalued due to the similarity of the histological characteristics of polyps with other more common clinical entities [5]. Therefore, gene testing in patients who meet Jass criteria can confirm the diagnosis. An inflammatory infiltrate and edematous lamina propria are the pathological features of juvenile polyps, along with dilated cystic glands that are lined by the foveolar epithelium and display reactive changes [6].

Germline SMAD4 and BMPR1A mutations on chromosomes 18 and 10, respectively, have been determined as the causative genes, with SMAD4 being predominant in patients with gastric involvement. SMAD4 is a tumor suppressor gene that mediates the TGFβ signaling pathway leading to apoptosis. Although the correlation between genotype and phenotype is still under research, SMAD4 mutations have been linked to gastric polyps, gastric cancer, and hereditary hemorrhagic telangiectasia (HHT) [2,7].

Gastric juvenile polyposis was first reported in 1979, and among the clinical manifestations that have been described, gastric outlet obstruction leads to progressive dilation of the stomach [8]. Compared to the aforementioned findings of our patient, the greatest gastric dilation ever documented is 60.8 cm along the greater and 24.2 cm along the lesser curvature in the context of massive gastric polyposis [9]. This case, to our knowledge, represents the largest documented gastric dilatation along the greater curvature, with implications for organ motility.

Juvenile syndromic polyps are a precancerous condition, with a reported risk of 40-50% and 21% for developing colorectal and gastric cancer, respectively [10]. Therefore, surveillance colonoscopy annually and upper GI endoscopy every one to three years is recommended to commence at 12 years old or earlier on the appearance of clinical manifestations. Polypectomy should be performed on polyps ≥5 mm, while indications for gastrectomy include high-grade dysplasia, gastric cancer, and massive gastric polyposis that cannot be managed endoscopically due to the high polyp burden [1].

## Conclusions

The aforementioned case report of gastric involvement in juvenile polyposis syndrome highlights the complexity arising from the potentially progressive multifocal manifestation of the syndrome. A multidisciplinary approach with clinical assessment, imaging, and genetic testing combined with a high index of clinical suspicion is required for optimal management of the patient. Therefore, consistent surveillance is required to address the precancerous nature and the heterogeneity of clinical manifestations of the syndrome.
